# Plasmonic Enhancement of Two-Photon Excited Luminescence of Gold Nanoclusters

**DOI:** 10.3390/molecules27030807

**Published:** 2022-01-26

**Authors:** Anna Pniakowska, Joanna Olesiak-Banska

**Affiliations:** Advanced Materials Engineering and Modelling Group, Wroclaw University of Science and Technology, Wybrzeże Wyspiańskiego 27, 50-370 Wroclaw, Poland; anna.pniakowska@pwr.edu.pl

**Keywords:** gold nanorods, gold nanoclusters, two-photon excited luminescence, single molecule detection, plasmonic enhancement

## Abstract

Plasmonic-enhanced luminescence of single molecules enables imaging and detection of low quantities of fluorophores, down to individual molecules. In this work, we present two-photon excited luminescence of single gold nanoclusters, Au_18_(SG)_14_, in close proximity to bare gold nanorods (AuNRs). We observed 25-times enhanced emission of gold nanoclusters (AuNCs) in near infrared region, which was mainly attributed to the resonant excitation of localized surface plasmon resonance (LSPR) of AuNRs and spectral overlap of LSPR band with photoluminescence of AuNCs. This work is an initial step in application of combined nanoparticles: gold nanorods and ultrasmall nanoclusters in a wide range of multiphoton imaging and biosensing applications.

## 1. Introduction

Unique optical properties of gold nanorods arise from collective oscillation of valence band electrons of metal nanoparticles, called surface plasmon resonance (SPR) [1]. Remarkable linear and nonlinear optical properties of gold nanostructures bring a wide range of application in surface-enhanced Raman spectroscopy [2], metal-enhanced fluorescence [3,4], and two-photon imaging [5]. Plasmonic nanostructures are one of the most commonly used materials as a platform of efficient optical detection for wide range of luminescent species [6,7,8,9]. Compared to other nanostructures, gold nanorods present narrow size distribution and well-established structure that lead to uniform optical properties, crucial for reliable investigation of single molecule and single-nanoparticle interaction [4,10]. Utility of gold nanorods is firmly motivated by tuneable surface plasmon resonance that can be tailored for the desired range of wavelengths above 600 nm. Besides, the tips of anisotropic plasmonic nanoparticles induce strong local electric field, easily accessible for interaction with chromophores floating nearby the nanoparticle. In this approach, amplification of electric field at the tips of nanorods led to enhanced luminescence of weakly emitting molecules. Careful selection of nanorods for interaction with specific fluorophores plays a key role in luminescence enhancement, since the mechanism of this process is strongly dependent on the spectral overlap of SPR band and emission spectrum of analysed fluorophores [6,11,12]. The distance between the nanoparticle and fluorophore is the second crucial factor governing emission enhancement [8,13]. Average highest enhancement has been observed in 5–20 nm distance from plasmonic surface [8,13,14]. 

Recent single-molecule findings provide detailed insights into processes that would be ordinarily averaged in bulk experiments. Although single-molecule studies of plasmon-enhanced luminescence detection are full of remarkable results discovered under one-photon excitation, studies of enhancement of multiphoton processes are still at the preliminary level. Compared to one-photon excitation, two-photon excitation is known for deeper penetration into (biological) samples, higher axial resolution, and better signal to noise ratio, therefore, it is preferred in bioimaging applications. Two-photon excited photoluminescence and other multiphoton processes can also benefit from plasmonic enhancement. In theory, with higher order of multiphoton processes, proportionally stronger enhanced responses may appear. However, enhancement on single gold nanoparticle requires strictly controlled femtosecond (fs) laser power to avoid photoinduced reshaping of nanoparticles [15,16]. Nonlinear investigation of plasmon enhanced luminescence of chromophores is a relatively new method, which was examined only with quantum dots [10,17], fluorescent dyes [12,18], or fluorescent proteins [19]. Nanorod-assisted two-photon processes were usually enhanced several times, e.g., 11-times for T790 dyes [12] and 4-times for PVP dyes [18], while single-molecule study presented extremely strong enhancement, e.g., 1000-times for eqFP670 protein [19] and 10,000-times for CdSe/ZnS QDs [10]. 

Gold nanoclusters (AuNCs) with overall particle dimensions below 2 nm were categorized as a new class of nanomaterials, which attracted remarkable attention in recent years. Their photophysical and optical properties, tailored by atomically precise structure, arise from discrete electronic structure and molecule-like behaviour [5]. Nanoclusters possess unique emission. Depending on the nanoclusters size and structure, it can be located from blue to near infrared (NIR), the most attractive region in terms of bioimaging applications. Recent studies on toxicity and biodistribution of Au_18_(SG)_14_ in in vivo systems show promising results and are the first step to utilizing nanoclusters for bioimaging and biosensing applications [20,21,22]. Nanoclusters are well-characterised under one-photon excitation, but also present excellent nonlinear optical properties, which can be utilized in two-photon imaging [23,24,25]. Gold nanoclusters were found to be remarkable two-photon absorbers with high 2PA cross-sections (several hundred GM to several hundred thousand GM) [26,27]. However, photoluminescence quantum yield of nanoclusters is usually very low. Therefore, many techniques are examined to enhance the luminescence of nanoclusters. 

Due to relatively low quantum yield of approximately 1–4%, it is challenging to image single nanoclusters using standard imaging techniques. To achieve single molecule sensitivity of detection, we used gold nanorod-assisted technique of luminescence enhancement of Au_18_(SG)_14_ (where SG stands for glutathione) on the basis of AuNCs diffusion in the vicinity of plasmonic nanoparticles. To determine most reproducible signal, we performed measurements on structurally consistent and equally sized nanorods and homogeneous nanoclusters build-up from the same number of gold atoms. In this manner, we synthesized and characterized one-photon optical properties of Au_18_ NCs, which were further investigated in nonlinear regime. We performed systematic study of optical signal of AuNRs-AuNCs system under two-photon microscope, imaging individual nanorods. We demonstrate 25-fold enhancement of two-photon luminescence of nanoclusters as a result of resonant excitation of plasmonic nanorods. 

## 2. Results

### 2.1. Synthesis and Characterisation of Gold Nanoclusters 

The glutathione-protected gold nanoclusters, Au_18_(SG)_14_, were synthesized on the basis of Ghosh protocol with minor modifications [28,29]. Sodium cyanoborohydride used here as a milder reducing agent than NaBH_4_ enabled the slowing down of the reduction of Au(I) and to favour size-selective growth of Au_18_(SG)_14_. The second factor influencing a slower reduction of gold is a dropwise addition of the reducer [29]. 

**Figure 1:**
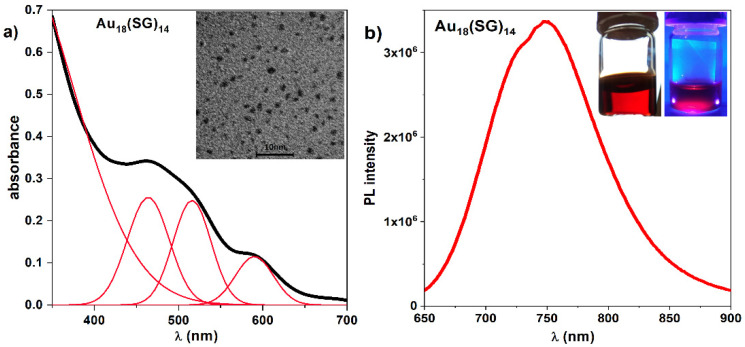
(**a**) Extinction spectra of Au_18_(SG)_14_ with marked bands at 465, 515, and 590 nm. Upper right corner presents TEM image of NCs. (**b**) Au_18_(SG)_14_ emission spectra with maximum peak position at 750 nm. Photographs in the inset present Au_18_(SG)_14_ cluster solution in visible (left) and UV light (right).

The extinction spectra of Au_18_(SG)_14_ obtained after synthesis revealed characteristic bands at 465, 515, and 590 nm (Figure 1a) and emission spectra at 750 nm (Figure 1b) following absorption and emission spectra reported previously [28,30]. The morphology of synthesized gold nanoclusters was determined under TEM (Figure 1a) revealing average size distribution equal to 1.75 ± 0.03 nm (Figure S1).

In order to determine purity and homogeneity of synthesized nanoclusters, polyacrylamide gel electrophoresis was applied along with parallel separation of Au:SG nanoclusters mixture, synthesized according to the protocol described in the materials and methods. Final product of both glutathione nanoclusters, atomically-precise Au_18_(SG)_14_ and Au:SG mixture, were maintained in a powder to provide better stability in time. In all experiments, the nanocluster powder was freshly dispersed in distilled water. Figure S2a shows well-distinguished fractions of Au:SG NCs marked 2–9, which correspond to nanoclusters with a precisely-defined number of building atoms: (2) Au_15_(SG)_13_, (3) Au_18_(SG)_14_, (4) Au_22_(SG)_16_, (5) Au_22_(SG)_17_, (6) Au_25_(SG)_18_, (7) Au_29_(SG)_20_, (8) Au_33_(SG)_22_, and (9) Au_39_(SG)_24_ [31]. The first fraction, Au_10_(SG)_10_, visible only under UV light, was omitted here. As-synthesized Au_18_(SG)_14_ NCs (right side of PAGE gel, Supplementary Materials, Figure S2a) shows only one band, indicating high purity of the synthesis. The same mobility of Au_18_(SG)_14_ NCs and third fraction of Au:SG mixture confirms identification of synthesized product. Visible discontinuity of the linear alignment of the PAGE fractions were explained by nonuniform polymerization of separating gel or local polymer overheating. Regardless, it does not deteriorate the highest precision of gel electrophoresis separation since each AuNCs fraction presents unique UV/VIS spectra, characteristic for particular types of atomically-precise nanocluster (Supplementary Materials, Figure S2b) that resemble these reported previously [31].

### 2.2. Single Nanorods Sample Preparation for Multiphoton Study

**Figure 2:**
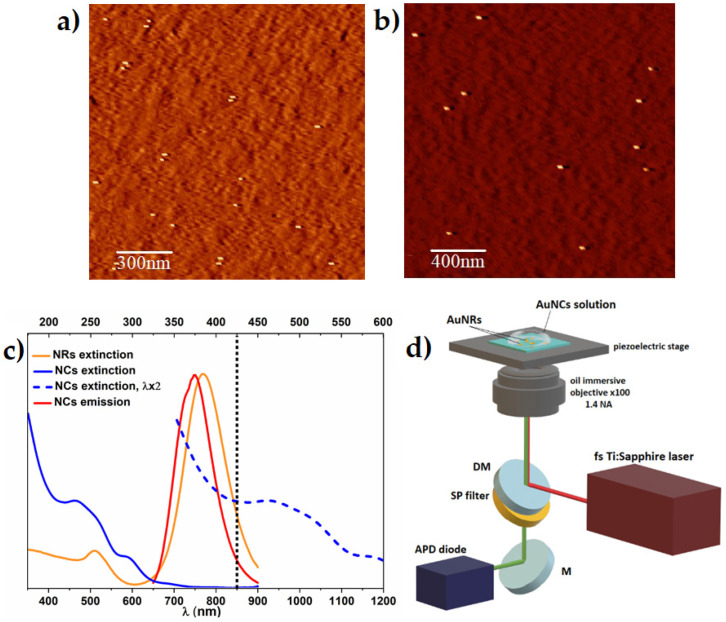
AFM images of (**a**) CTAB-coated and (**b**) bare gold nanorods. (**c**) Spectral overlap of longitudinal surface plasmon resonance band of nanorods (orange) and emission band of Au_18_ nanoclusters emission (red). One-photon absorption of nanoclusters (blue solid line) with expected two-photon absorption spectrum (blue dotted line), plotted as one-photon absorption at the double wavelength. Vertical black dotted line represents the laser wavelength used in two-photon excitation (λ_exc_ = 850 nm). (**d**) A scheme of the experimental setup (NA = numerical aperture, APD = avalanche photodiode, DM = dichroic mirror, and M = mirror). Above the objective and on the top of the piezoelectric stage, glass sample is placed with immobilised gold nanorods and a droplet of nanoclusters diffusing in a glycerol solution.

Plasmonic enhancement of two-photon-excited luminescence was carried out on carefully prepared substrate of well dispersed nanoparticles acting as resonant platform for floating fluorophores. Gold nanorods used in this work present narrow size distribution within the sample, with average size of 42.2 ± 7.6 nm × 17.4 ± 3.8 nm, as determined by AFM microscope. AFM images of both samples, with 0.84 ug/mL concentrated AuNRs with CTAB, and with lowered concentration of CTAB, show relevant separation of nanoparticles, essential for further multiphoton study (Figure 2a,b). We determined ≈11 nm length difference between covered and bare gold nanoparticles, implying ≈5.5 nm thick CTAB bi-layer (Supplementary Materials, Figure S3), which is in good agreement with the literature [32,33]. Dark-field images and single molecule scattering spectra of individual AuNRs present overlapping LSPR peak position as overall solution of AuNRs, indicating high homogeneity of plasmonic nanoparticles (Supplementary Materials, Figure S4).

### 2.3. Multiphoton Study of Single Nanoclusters Luminescence Enhancement on Single Nanorods

To verify wavelength dependence of two-photon absorption of AuNCs, we examined two-photon excited luminescence (TPEL) at several wavelengths and confirmed the origin of photoluminescence in simultaneous absorption of two photons for λ > 820 nm (see a log-log plot of TPEL intensity as a function of the incident laser average power in Supplementary Materials, Figure S5). Multiple reports [6,8,11] established that the strongest plasmonic enhancement of fluorophore emission was observed when excitation wavelength match the maximum of LSPR wavelength. To efficiently excite both the longitudinal surface plasmon resonance of AuNRs (λ_LSPR_ = 770 nm) and AuNCs in their range of two-photon absorption, we moved excitation wavelength to the NIR region to 850 nm (Figure 2c). The two-photon luminescence enhancement experiments were performed as shown in Figure 2d, with optical set-up described in detail in materials and methods.

**Figure 3:**
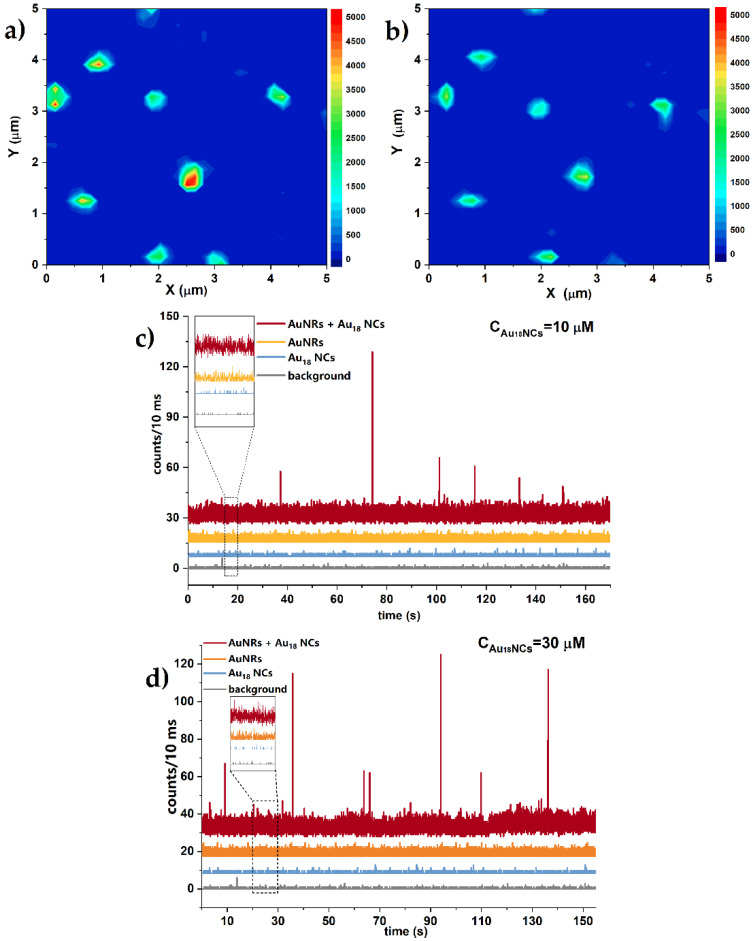
(**a**,**b**) TPL intensity map of well-separated AuNRs before (**a**) and after (**b**) covering with glycerine solution of AuNCs. (**c**,**d**) luminescence time traces of samples: AuNRs with local emission enhancement of floating AuNCs (red), solutions of AuNRs only (orange), AuNCs only (blue), and background noise (grey), monitored under prolonged irradiation of 70 μW in 10 ms photon counting window. AuNCs emission enhancement was determined in two concentration dependent conditions: (**c**) 10 μM and (**d**) 30 μM.

Figure 3a shows multiphoton luminescence intensity images obtained by scanning the sample of gold nanorods immobilized on a glass surface. Individual bright spots correspond to strong emission intensity of well-dispersed single AuNRs. Stable photoluminescence from a single plasmonic emitter was recorded under prolonged laser power of 70 µW at the beam focus, as presented on the emission time trace graph (Figure 3c,d). Single gold nanorod maintained constant emission intensity for at least 3 min, which indicated preserved original shape of a nanorod (Figure 3c,d yellow lines). Chosen laser power was sustained through all measurements as it was not harmful for nanorods, yet strong enough to detect single nanoparticle luminescence. The photon count window (bin time) was set to 10 ms to register clear on/off states of enhanced luminescence, not disturbed by signal to noise ratio. 

Gold nanorods immobilised on a glass plate were further covered by Au_18_(SG)_14_ solution droplet (1:1, H_2_O:glycerine). TPEL intensity scan repeated in exactly the same location of the sample shows unchanged nanorods distribution, indicating good adhesion of bare nanorods to the glass coverslip (Figure 3b). Negligibly lower TPEL intensity after addition of nanoclusters glycerol solution was related with the change of dispersion medium and respective refractive index. TPEL time traces from single gold nanorods show constant TPEL signal with frequent enhanced luminescence bursts from floating AuNCs in the vicinity of plasmonic nanoparticles, that last for 10–30 ms (Figure 3c,d, red line). To distinguish recorded emission enhancement of AuNCs–AuNRs hybrid from reference signals, we conducted TPEL time traces of separate components: single AuNRs, solution of AuNCs, and the background noise in the same conditions. Size limitations of luminescent techniques exclude the detection of a single gold nanocluster without plasmonic-mediator, thus, we determined the lowest detectable concentration of nanoclusters to be 10 μM at the strongest power illumination (limited by melting and reshaping of nanorods). In these conditions, ≈6 single nanoclusters were illuminated at the same time. Therefore, we report up to 25-times stronger luminescence of single Au_18_ NCs in the close proximity of a NR, compared to TPEL of NCs solution with low concentration of NCs, measured without the presence of gold nanorods.

**Figure 4:**
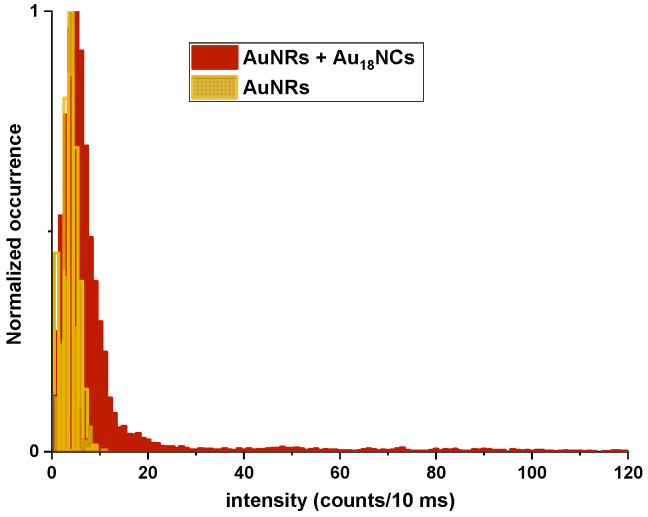
Distribution of number of occurring events of gold nanoclusters TPEL with gold nanorods (red) and gold nanorods itself (yellow).

Calculated average number of occurring events addresses the difference between monitored intensity of AuNCs + AuNRs sample vs. AuNRs alone (Figure 4). To confirm that the observed luminescence bursts are associated with the presence of nanocluster molecules in close proximity of nanorods, we conducted additional experiments of emission time traces in identical conditions, exchanging nanocluster molecules with pure water:glycerol solution. As expected, no luminescence enhancement occurred in this circumstance [Supplementary Materials, Figure S6]. 

Establishing the conditions of AuNCs concentration and viscosity are crucial for studies of the photoluminescence intensity enhancement in the vicinity of nanoparticles. We performed two-photon excited luminescence analysis of AuNCs at two concentrations, C = 10 µM, 30 µM, and in different water:glycerine content. The most frequent enhancement events were obtained for 30 µM Au_18_(SG)_14_ diluted in 1:1 solution of water and glycerine. High viscosity of glycerine slows down the overall movement of single nanoclusters, while pure glycerine generates too high adhesive tensions with the glass surface, which leads to peeling of single nanorods from the support. A 1:1 solution of water and glycerine has the advantage of glycerine usage without causing any dislocation of AuNRs.

## 3. Discussion 

The first requirement of the investigation of the single-molecule two-photon-excited luminescence is appropriate preparation of single nanoparticles sample as an emission enhancement platform for floating fluorophores. Gold nanorods capped with CTAB surfactant stabilize the water suspension, therefore drop-casting the solution of nanoclusters on the top of immobilized nanorods conveyed the nanorods from the surface of glass sample. Removal of CTAB layer resulted in raised surface tension and stronger adhesion of bare nanorods to the surface [34]. Additionally, by decreasing the amount of positively charged CTAB on the surface of nanoparticle, the overall electric charge of nanoparticles is closer to neutral [35,36]. It creates better conditions for free diffusion of negatively charged AuNCs in a close area of nanorods, without permanent attachment. Removed CTAB layer enabled monitoring of molecule interaction at closest possible distance. Although the literature findings specify the strongest luminescence enhancement at 10–20 nm between metal surface and fluorophore [8], similar to W. Zhang [37] and E. Wientajes [38], we observed more frequent and more intense bursts of luminescence when AuNCs interacted with bare single nanorod at the shorter distance. It finds good agreement with a particular study, where plasmonic assisted two-photon luminescence occurred at shorter distance of interactions than one-photon ones. The closer the excitation was to the surface plasmon resonance band, the stronger the local electric enhancement, and therefore, stronger two-photon excited luminescence was monitored. Moreover, two-photon excitation process involves simultaneous absorption of two photons, and thus, the excitation rate is proportional to the fourth power of local electric field [12].

Monitored 25-fold plasmonic luminescence enhancement of Au_18_ single molecules might reach higher values, when compared to single Au_18_ nanoclusters. Nevertheless, monitored enhancements highlight the benefit of single-particle luminescence detection over the average signal of gold nanoclusters bulk solution. However, to study emission enhancement of single molecules, the crucial part is the choice of excitation conditions for monitoring two-photon excited luminescence. The most intensive single molecule luminescence was mainly observed at resonant excitation of plasmonic nanoparticle. Plasmon peak placed between the absorption and emission maximum of fluorophore fulfil the requirements of strongest emission enhancement [39]. In this work, we established conditions for efficient excitation of both longitudinal surface plasmon resonance of AuNRs (λ_LSPR_ = 770 nm) and AuNCs in their range of two-photon absorption (λ > 820 nm). Since chosen resonance excitation wavelengths have major impacts on the final result of luminescence enhancement, shifting of the laser wavelength around 50 nm from the most favourable region of LSPR maximum may lead to more than one order of magnitude in dropped luminescence enhancement [10]. Therefore, we assume that multiphoton luminescence of AuNCs may potentially reach higher values than reported in this work, with 25-fold enhancement at λ_exc_ = 850 nm. However, we highlight here the requirements of overlap of LSPR and emission of nanoclusters in NIR region. 

Random distribution of enhanced TPEL signal above average 6 counts/10 ms of AuNRs, up to 120 counts/10 ms (Figure 4), was attributed to the AuNCs in close proximity of nanorods and may be initiated by several factors. Different level of enhancement of chromophores were previously explained by random orientation of molecules in the resonance field of nanorods [10]. Au_18_ NCs presents core-shell elongated structure, as shown on the structure model in Figure S7 [40]. Therefore, we assume that anisotropy of AuNCs structure results in enhancement dependent on the mutual orientation of NCs and a nanorod which may affect final luminescence burst intensities, while freely floating AuNCs near plasmonic particle are in different local position. Moreover, the strongest enhancement of emission signal was monitored at the sharp ends of nanorods, in the strongest local electromagnetic field [4,41]. Thus, free motion of AuNCs around a nanorod allow local interaction, with the tip and lateral side of plasmonic nanoparticle inducing different level emission enhancement proportional to the near-field intensity of a nanorod. 

According to W. Zhang [37], when two molecules interact with the same nanorod at the same time, luminescence signal will reach another higher level of enhancement. Although the average <2 nm size of nanoclusters creates space for simultaneous collective action of luminescence enhancement, the experiment was carried with low concentrated AuNCs to prevent multiple circumstances that might disturb single-molecule investigation. Recorded low number of occurrences excludes the possibility of several actions at the same time. Short duration of single bursts in the order of 10 ms confirms the presence of a single nanocluster in the hotspot during local enhancement. Similar duration of emission enhancement of freely diffusing single molecule in the area of a gold nanorod was previously noted [42]. There were two solutions to preserve molecules for longer in the vicinity of near-field of nanorods: raise the viscosity of solvent, or use additional linkers [37,42]. We examined plasmonic enhancement of nanoclusters in solvents of the range of different densities, however, except of slower diffusion of fluorophores, high glycerine content solvents peel off immobilised nanorods from a glass surface. We report in this work optimised conditions of slow free diffusion for single nanocluster detection. 

We highlight the importance of strictly controlled femtosecond laser power in multiphoton studies, since intensive laser source heats up the plasmonic electron gas that induces nanoparticle reshaping, broadening, and/or shift the plasmon resonance [15,16,43], and significantly lower plasmon-mediated enhancement. In this work, we performed multiple experiments on nanorods stability under prolonged fs laser illumination. Chosen average power (70 μW) did not affect nanoparticles structure, assuring the reproducibility of enhanced luminescence results. Repeatable experiments of two-photon excited luminescence of gold nanoclusters assure future standardization of processes and usability in bio-detection. Technique of plasmon-enhanced luminescence of single molecules already has its first successful bioimaging application in lipid membrane, a model system for studying biological membranes [44]. Application of two-photon excitation ensures strong fluorescence enhancement for single-molecule imaging of cells [12]. Additional benefits of two-photon excitation over one-photon one, e.g., lowered sample photodamage and deeper penetration across the sample are strongly desirable for imaging and sensing applications in near infrared region.

## 4. Materials and Methods

### 4.1. Chemical

All the chemicals are commercially available and used without further purification. Gold(III) chloride trihydrate (HAuCl_4_·3H_2_O, 99.999%), L-Glutathione reduced (GSH) ≥ 98.0%, sodium cyanoborohydride (NaBH_3_CN, 95%), sodium borohydride (NaBH_4_, 99.99%), and aqueous suspension of gold nanorods (10 nm diameter, 780 nm SPR absorption maximum) were purchased from Sigma-Aldrich. Aqueous 40% acrylamide and bisacrylamide stock solution (37.5:1), N,N,N′,N′-tetramethylethylenediamine (TEMED, 99%), N,N′-methylene bisacrylamide (Bis, >98%), and 10× concentrated SDS-PAGE running buffer (SDS) were supplied from Carl Roth. Ammonium persulfate (APS, 98%), 0.5 M Tris-HCl buffer pH 6.8, and 1.5 M Tris-HCl buffer pH 8.8 were purchased from BIO-RAD. Deionized (milli-Q) water with a resistivity of 18 MΩ cm was used in this work. 

### 4.2. Synthesis of Gold Au_18_(SG)_14_ Nanoclusters

Synthesis of nanoclusters was based on the protocol given by Ghosh [28], with further modifications reported by Manzhou Zhu [30] and Stamplecoskie [29]. Briefly, 0.6 mL of MeOH, 0.6 mL of water, and 150 mg of GSH were gently mixed in 25 mL round flask for 10 min. Then, 0.3 mL aqueous solution of HAuCl_4_·3 H_2_O (0.635 M, 75 mg) was added. Colour of stirred solution slowly changed from yellow to almost colourless, indicating the conversion of Au^3+^ to Au^+^. After 10 min, solution was diluted to 15 mL by MeOH, followed by slow dropwise addition of 2.25 mL methanolic solution of NaBH_3_CN (220 mM). After 2 h of vigorous stirring, the precipitate was collected and washed with MeOH three times through centrifugal precipitation (10 min, 7000 rcf). Then, solution was dissolved in a small amount of water, centrifuged to remove unreacted thiolate, and finally dried to obtain red powder.

### 4.3. Synthesis of Gold Au:SG Nanoclusters

Synthesis of gold Au:SG nanoclusters followed the protocol by Tsukuda and co-workers [31]. First, 25 mL of MeOH was carefully stirred with 153 mg of GSH for 10 min in 50 mL round flask. Then, 0.197 mL of aqueous solution of HAuCl_4_·3H_2_O (0.635 M) was added, stirred until mixture became almost colourless, and cooled down in cool bath for 30 min. Next, freshly prepared cold NaBH_4_ (6.25 mL, 0.2 M) was rapidly injected into vigorously stirred mixture. Instant change of colour to dark brown implied fast reduction of gold to Au^+^. After 1 h of vigorous stirring, precipitate was collected and washed repeatedly with MeOH through centrifugal precipitation (10 min, 7000 rcf) to remove the remaining precursors. Then, solution was left to complete evaporation of solvent. 

### 4.4. PAGE Electrophoresis

A separation of the clusters was performed according to the procedure of polyacrylamide gel electrophoresis (PAGE) [45,46]. Detailed volumes are collected in Table S1 (Supplementary Materials). The separating and stacking gels were prepared from acrylamide monomers with the final concentrations of 24.3%T; 3.8%C of high density gel and 20%T; 2.6%C of low density gel, where %T denotes the total monomer (acrylamide and bisacrylamide) concentration and %C is the concentration of the crosslinking. The clusters were dissolved in 5% (*v*/*v*) glycerol/water to final concentration of 5 mg/mL. A 1 mm gel thickness was used for preparative separations into cluster fractions. Electrophoresis was carried out for 16 h in a constant voltage mode set to 150 V. Finally, the separating gel was cut into stripes, and the particular fractions were eluted with water.

### 4.5. Preparation of Gold Nanorods Sample and Imaging of Gold Nanoparticles

Aqueous suspension of gold nanorods purchased from Sigma Aldrich were stabilized by cetyltrimethylammonium bromide (CTAB). To immobilize nanoparticles on the glass coverslip and provide access of floating fluorophores on the surface of bare gold nanorods, an outer layer of CTAB had to be removed. For this purpose, a small amount (50 μL, 30 μg/mL) of the nanoparticles solution was washed with deionized water and centrifuged (300 rcf, 3 min) several times until gold nanorods were still able to suspend in water without permanent aggregation. Such prepared bare gold nanorods in deionized water were deposited by drop casting on purified glass coverslip. After 10 min, the droplet was washed out and air dried. Same procedure was performed for a set of several concentrations (4.2 μg/mL, 0.84 μg/mL, and 0.084 μg/mL). Then, gold nanorods separation and size distribution on glass coverslip were estimated with Atomic Force Microscope (AFM) (Dimensional V scanning probe microscope, Veeco) operating in a tapping mode. Nanoparticles separation was further confirmed with dark field imaging using Nikon Eclipse inverted optical microscope with Nikon Dark Field Condenser. Scattering spectra were recorded using a Shamrock 303i spectrograph from Andor. 

The morphological features of the AuNRs and AuNCs were determined using FEI Tecnai G2 20 X-TWIN transmission electron microscopy (TEM).

### 4.6. Characterization of Linear and Nonlinear Optical Properties of Nanoparticles

Extinction and emission spectra of the synthesized gold nanoclusters and nanorods were obtained using a JASCO V-670 Spectrophotometer and Hitachi F-4500 spectrofluorometer.

Multiphoton experiments of both AuNRs and AuNCs were conducted under custom-made setup. The glass samples were placed on the XYZ piezo-electric scanning stage (TRITOR 102, Piezojena) and excited using a tuneable, mode-locked Ti:sapphire laser operating at 80 MHz pulse repetition rate (Chameleon, Coherent Inc., Santa Clara, CA, USA). A high numerical aperture Nikon Plan Apo oil immersion objective (100×/1.4 NA) was used for the focusing of a circularly polarized incident laser beam on the sample and for the collection of multiphoton-excited emission in epifluorescence mode. The emitted signal was separated from the incident laser beam on a dichroic mirror and collected by avalanche photodiodes operating in the photon counting regime. The two-photon excited emission spectra were recorded using a Shamrock 303i spectrograph from Andor.

## 5. Conclusions

In conclusion, our goal was usage of plasmonic nanoparticles to detect gold nanoclusters on the single-molecule level, which is challenging to pursue in conventional techniques. We proved the advantage of single-molecule technique of detection, emphasizing the difference in the emission intensity of single particles against the average signal of a bulk form of nanoparticles. We conduct, for the first time, the enhancement of weakly emissive atomically-precise nanoclusters on a single plasmonic nanoparticle, under two-photon excitation. Wet-chemically synthesised nanoclusters, Au_18_SG_14_, present 25-times stronger two-photon excited luminescence in close proximity of homogeneous single nanorods by the strong local field of a nanorod under surface plasmon resonance excitation. TPEL enhancement was addressed to well-selected excitation conditions: two-photon excitation of gold nanoclusters at the wavelength between excitation of LSPR of the plasmonic nanoparticle and emission of nanoclusters. Two-photon absorption of nanoclusters was confirmed by quadratic dependence of TPEL of nanoclusters on average power of excitation laser.

Our preliminary study of single nanocluster–single plasmonic nanoparticle interactions provides new opportunities for gold nanoclusters applications in single particle sensing and imaging. We reveal in this work that nanoclusters may find potential application as ultrasmall fluorophores in multiphoton bioimaging and biosensing, with particular interest of luminescence in near-infrared region.

## Data Availability

Data is contained within the article or Supplementary Materials.

## Supplementary Materials

The following supporting information can be downloaded. Figure S1: (a) TEM image and (b) size distribution histogram of Au_18_(SG)_14_ nanoclusters.; Table S1: Page electrophoresis; Figure S2: (a) Photograph of electrophoretic polyacrylamide gel with well-separated 2-9 fractions of Au:SG mixture (left) and single fraction of Au_18_(SG)_14_ product of synthesis (right), which after electrophoresis separation were cut into pieces and dissolved in water (below). (b) Extinction spectra of 2–9 fractions of Au:SG mixture. (c) Comparison of UV/VIS spectra of as-synthesized Au_18_(SG)_14_ NCs and 3rd fraction of Au:SG mixture (identified as Au_18_ NCs) from PAGE separation.; Figure S3: Size distribution of nanorods before and after CTAB layer removal. Average diameter of NRs with CTAB layer is estimated as 42.2 ± 7.64 nm, while NRs without CTAB layer is 31.2 ± 8.22nm.; Figure S4: (A) Dark field image of gold nanorods well-separated on the glass sample. (B) Extinction spectrum of gold nanorods solution and scattering spectrum (red line) of single gold nanorod.; Figure S5: Log-log plot of the PL intensity of AuNCs as a function of excitation laser power measured at several excitation wavelengths. The slope of the linear fit to each datapoints sets corresponds to the level of multiphoton processes, n (for two-photon absorption n = 2).; Figure S6: Luminescence time traces of samples: AuNRs in water: glycerine solution in the absence of AuNCs (red). Samples are compared to AuNRs only (orange) or AuNCs solution only (blue) and background noise (grey), monitored under prolonged irradiation with average power 70 μW.; Figure S7: 3D model of Au_18_(SG)_14_ structure with precise location of gold core (yellow—Au atoms) and staple motifs (yellow—Au atoms, red-S atoms) of nanocluster. Structure was energy-minimized with molecular mechanic MM2 model in Chem3D. References [29,29,47,48] are cited in the supplementary materials.

## Sample Availability

Not available.
