# Supplementary material for: Plasmonic Enhancement of Two-Photon Excited Luminescence of Gold Nanoclusters
Source: Molecules. 2022 Jan 26;27(3):807. doi: 10.3390/molecules27030807 (PMC8838299; doi:10.3390/molecules27030807)
Supplement: Supplementary file 1 [file molecules-27-00807-s001.zip › molecules-1557818-supplementary.pdf]

molecules MDPI

Plasmonic Enhancement of Two-Photon Excited Luminescence of Gold Nanoclusters

Anna Pniakowska, Joanna Olesiak-Banska

SUPPLEMENTARY MATERIALS

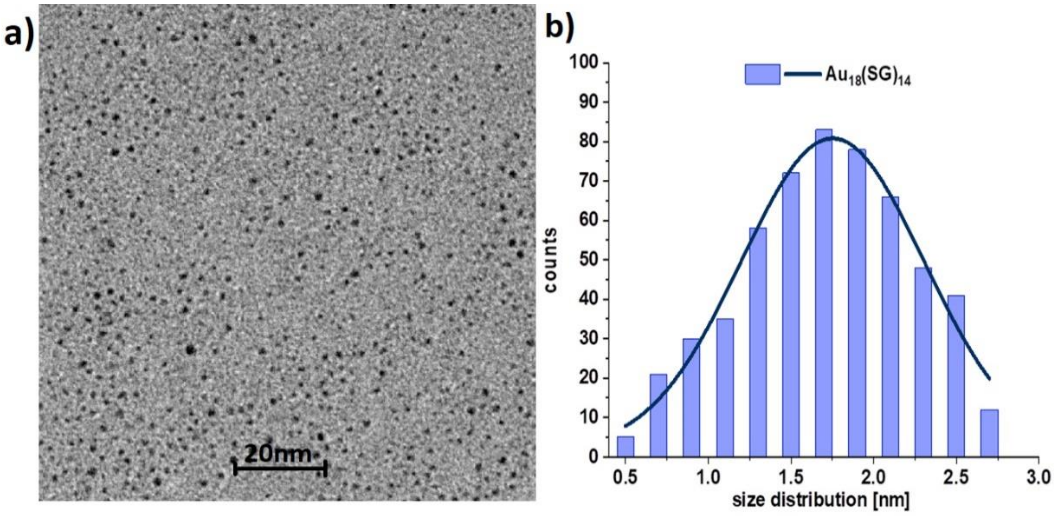

Figure S1. a) TEM image and b) size distribution histogram of $Au_{18}(SG)_{14}$ nanoclusters.

Table S1. Page electrophoresis

Reagent	Low density (LD) 20%T, 2.6%C	High density (HD) 24.3%T; 3.8%C
Monomer	25mL	72mL
N,N′-methylene bisacrylamide	-	18mL
Distilled water	12.5mL	18mL
1.5M Tris-HCl	12.5mL	30mL
10% APS	250µL	300 µL
TEMED	25µL	30µL

[a]40% total monomer (acrylamide+bis-acrylamide) stock solution (37.5:1)

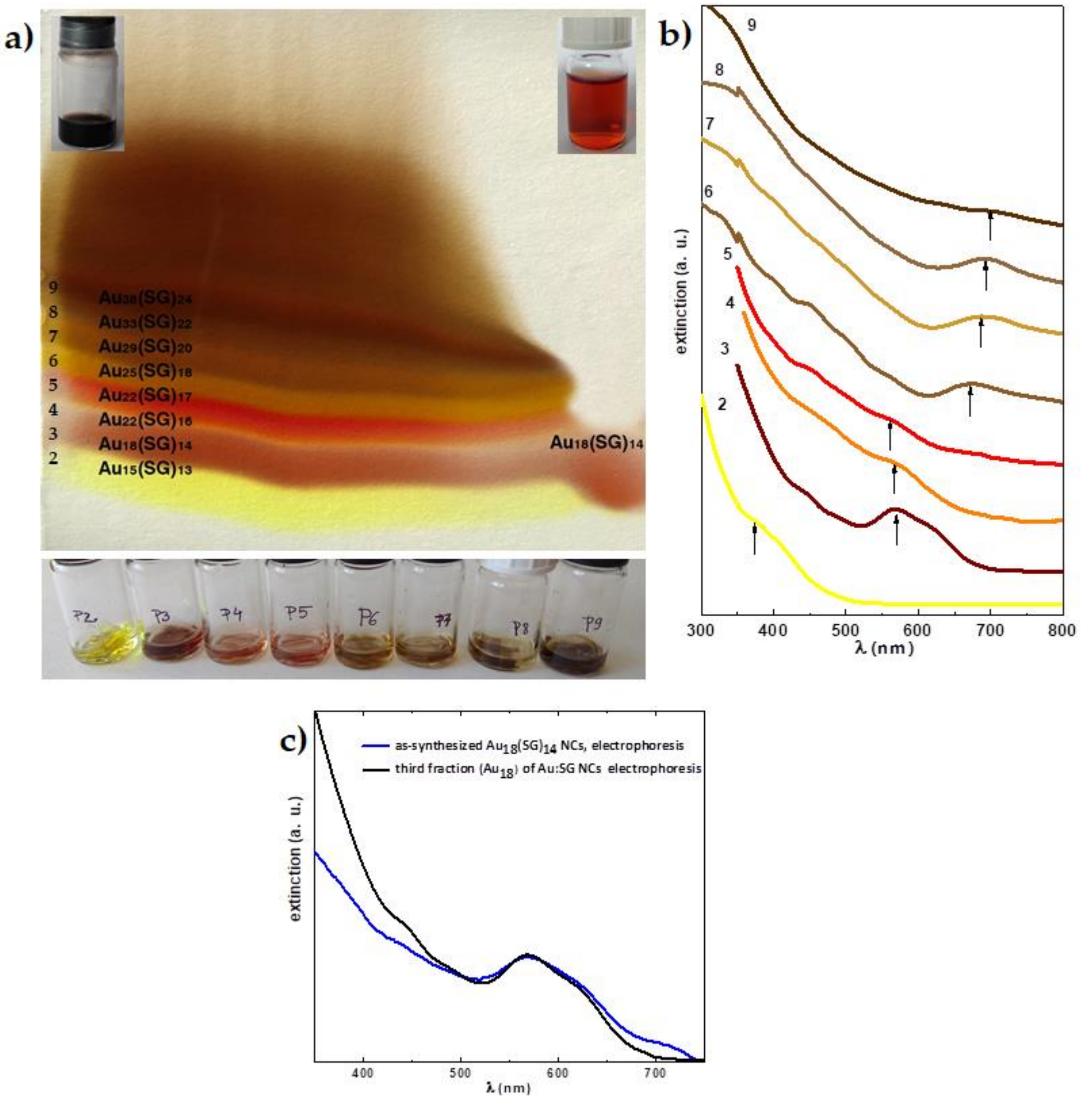

Figure S2. a) Photograph of electrophoretic polyacrylamide gel with well-separated 2-9 fractions of Au:SG mixture (left) and single fraction of $Au_{18}(SG)_{14}$ product of synthesis (right), which after electrophoresis separation were cut into pieces and dissolved in water (below). b) Extinction spectra of 2-9 fractions of Au:SG mixture. c) Comparison of UV/VIS spectra of as-synthesized $Au_{18}(SG)_{14}$ NCs and 3rd fraction of Au:SG mixture (identified as Au_{18} NCs) from PAGE separation.

Off note: electrophoresis induce changes in the structure of nanoclusters, therefore the spectra of nanoclusters after separation are shifted around 110 nm with respect to spectra of initial AuNCs. Yet, both absorption spectra of as-synthesized $Au_{18}(SG)_{14}$ and shifted third fraction of Au:Ag (fig. S2c) are identified as Au_{18} NCs in other sources. [1-4]

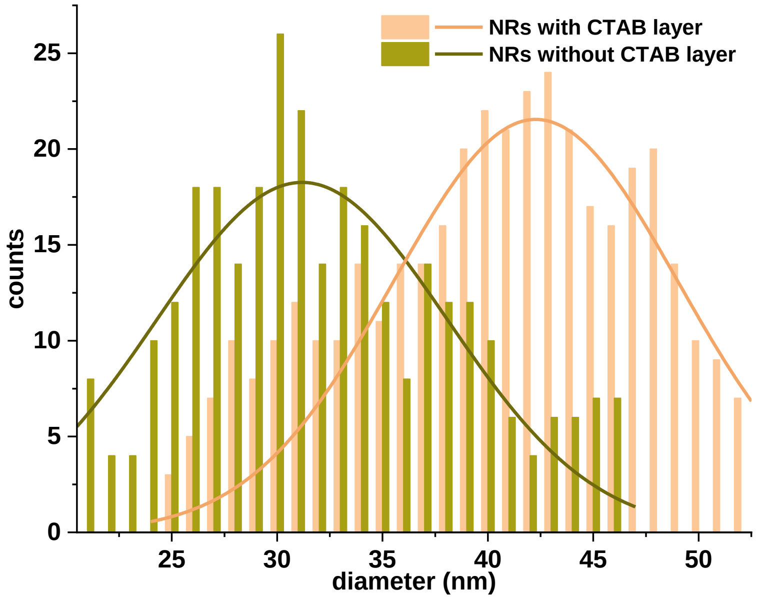

Figure S3. Size distribution of nanorods before and after CTAB layer removal. Average diameter of NRs with CTAB layer is estimated as 42.2 ± 7.64 nm, while NRs without CTAB layer is 31.2 ± 8.22nm.

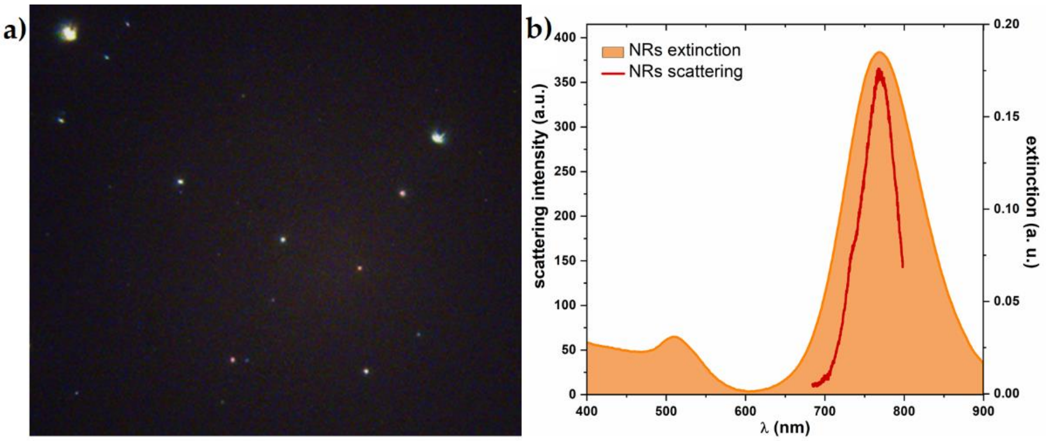

Figure S4. A) Dark field image of gold nanorods well-separated on the glass sample. B) Extinction spectrum of gold nanorods solution and scattering spectrum (red line) of single gold nanorod.

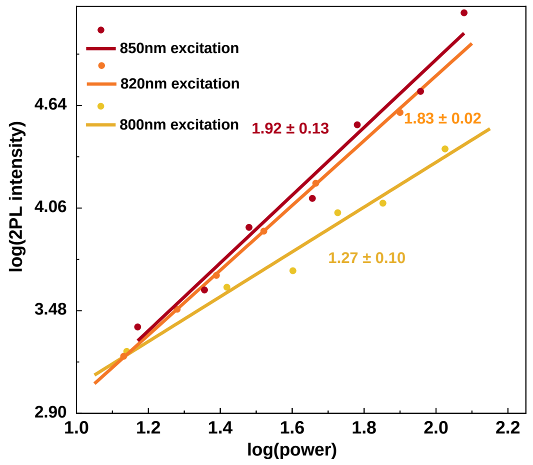

Figure S5. Log-log plot of the PL intensity of AuNCs as a function of excitation laser power measured at several excitation wavelengths. The slope of the linear fit to each datapoints sets corresponds to the level of multiphoton processes, *n* (for two-photon absorption n = 2).

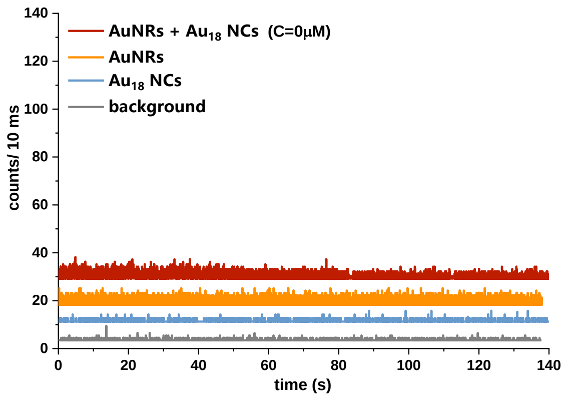

Figure S6. Luminescence time traces of samples: AuNRs in water: glycerine solution in the absence of AuNCs (red). Samples are compared to AuNRs only (orange) or AuNCs solution only (blue) and background noise (grey), monitored under prolonged irradiation with average power 70 μW.

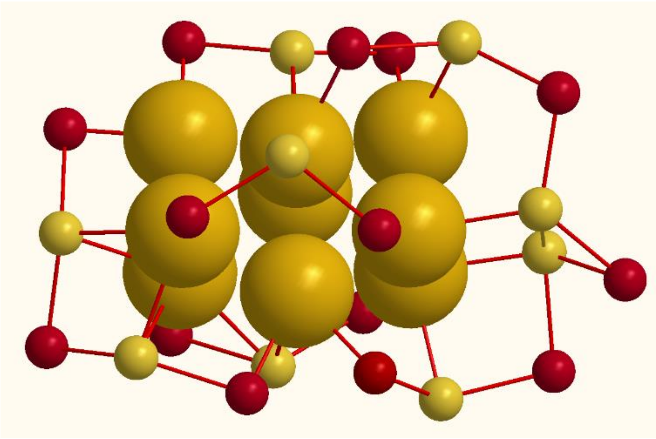

Figure S7. 3D model of $Au_{18}(SG)_{14}$ structure with precise location of gold core (yellow – Au atoms) and staple motifs (yellow – Au atoms, red-S atoms) of nanocluster. Structure was energy-minimized with molecular mechanic MM2 model in Chem3D.

1. A. Ghosh, T. Udayabhaskararao, T. Pradeep, One-Step Route to Luminescent $Au_{18}SG_{14}$ in the Condensed Phase and Its Closed Shell Molecular Ions in the Gas Phase. *J. Phys. Chem. Lett.* **2012,** *3*, 1997–2002.
2. Stamplecoskie, K. G.; Chen, Y.-S.; Kamat, P. V., Excited-State Behavior of Luminescent Glutathione-Protected Gold Clusters. *The Journal of Physical Chemistry C* **2014,** *118* (2), 1370-1376.
3. Jin, R., Atomically precise metal nanoclusters: stable sizes and optical properties. *Nanoscale* **2015,** *7* (5), 1549-1565.
4. Yu, Y.; Yao, Q.; Chen, T.; Lim, G. X.; Xie, J., The Innermost Three Gold Atoms Are Indispensable To Maintain the Structure of the Au18(SR)14 Cluster. *The Journal of Physical Chemistry C* **2016,** *120* (38), 22096-22102.
